# Effects of tissue-specific biomolecules on piglets after-weaning period

**DOI:** 10.14202/vetworld.2021.168-175

**Published:** 2021-01-21

**Authors:** Ekaterina Romanovna Vasilevskaya, Liliya Vyacheslavovna Fedulova, Irina Mikhailovna Chernukha, Elena Alexandrovna Kotenkova, Angelina Igorevna Fokina

**Affiliations:** V. M. Gorbatov Federal Research Centre for Food Systems of RAS, Moscow, Russia

**Keywords:** biomolecules, health, immune system, pig, sustainable pork production

## Abstract

**Background and Aim:**

Now-a-days antibiotics are the main tool for correcting the pathological conditions of pigs; unfortunately, antibiotics are a potential threat to the environment, as they lead to the spread of antibiotic-resistant infections. This study aimed to study the immunomodulatory encapsulated biomolecules on piglets in the post-weaning period.

**Materials and Methods:**

An immunomodulator based on biomolecules obtained from animal raw materials included in alginate capsules to improve absorption has been developed. The study presents the results of a study on 25 weaned piglets (25-30 days old) which received biomolecules at a dose of 200 mg/piglet for 14 days, followed by 400 mg/piglet from days 15 to 28. Blood was taken from animals for analysis (biochemical, hematological, cytometric, and enzyme immunoassay) and the integral index of blood serum antimicrobial activity was determined.

**Results:**

Experimental animals, whose initial weight was 1.6 times less than that of the control animals, were able to bridge this gap and, on the 28^th^ day, there were no differences in weight. Stimulation of the production of cytokines interleukin (IL)-2 and IL-4 was observed and the antimicrobial resistance of blood serum to *Escherichia coli* also increased. A positive effect on the metabolism of piglets was noted, which helped them adapt to a change in diet (from colostrum to solid food).

**Conclusion:**

The results show that the immunomodulation at the dose of 150 mg/kg body weight has a great potential for improving weaned pigs.

## Introduction

One of the basic critical needs of veterinary medicine and animal husbandry is prevention and control of the spread of existing and newly emerging pathogens. Treatment and preventive measures aimed at reducing the risk of infection with infectious diseases traditionally include vaccination and antibiotic therapy, including feed antibiotics [1]. The use of antibiotics in animal husbandry, as a panacea, leads to an increase in the cases of microorganisms with multiple resistance that has not even had direct contact with antibiotic agents, due to the acquisition of resistance genes (r-genes and plasmids) [2-4]. It is worth noting that even if a specific antibiotic is no longer introduced into the environment, r-genes are stored in bacteria, which have since been replicated without continuous exposure [5,6]. Thus, the inappropriate use of antibiotics in animal husbandry entails the accumulation in the surrounding section of highly resistant pathogens, mostly epizootically not relevant, but is latent threat of emergent infections that are dangerous for both animals and humans. In addition, treatment of infectious diseases that arise by transmission of resistant pathogens to people through a polluted environment and agricultural products is complicated by the lack of response to treatment with modern chemotherapeutic agents [7].

In recent years, the main trend of the world scientific community is the search for effective and safe natural drugs with anti-inflammatory and immunomodulatory effects, which are aimed at increasing the body’s resistance and enhancing the immune system [8-10].

We have shown the possibility of creating a new class of drugs – non-specific immunity stimulants, based on tissue- and species-specific biomolecules isolated from *Sus scrofa* immune organs using water with a modified isotopic (D/H) composition as a solubilizing agent [11,12]. The proteomic studies performed allowed us to identify more than 20 functional proteins in the spectrum of the isolated biomolecules, which are included in the immune response mechanism, exhibiting antioxidant. and anti-inflammatory properties, as well as stimulating metabolic processes in the body [13]. Efficacy of the resulting biomolecule complex has been shown by *in vivo* experiments on laboratory rats using a cyclophosphamide-induced immunodeficiency model. The main mechanism was stimulation of the production of cytokines by thymic immune cells as well as production of antibodies by B-system cells. Furthermore, an increase in non-specific resistance as well as in the adaptive capacity of productive animals were also noted [14,15].

Weaning of piglets from the sow is practiced in pig farming to intensify production. The pronounced consequences of early weaning and at low body weight (BW) are inhibition of growth, a decrease in the body’s resistance and a sharp increase in morbidity, which lead to significant death of animals [16]. This study aimed to examine the immunomodulatory potential of encapsulated biomolecules on piglets in the post-weaning period.

## Materials and Methods

### Ethical approval

The experimental protocols used in the current study were reviewed and approved by the Bioethical Committee of V. M. Gorbatov Federal Research Centre for Food Systems of RAS, Moscow, Russian Federation (2/2019).

### Study period and location

The experiment was conducted at the V. M. Gorbatov Federal Research Centre for Food Systems of RAS, Moscow, Russian Federation (55°43’53”N, 37°40’44”E) during the period from October 10 to November 20, 2019.

### Isolation of bioactive components

Bioactive components, including proteins and peptides, were obtained as previously described [15]. The technology includes extraction of *Sus scrofa* thymus, spleen, and lymph nodes by water-salt solution (0.9% NaCl) containing 50 ppm deuterium, after which the extracts are purified and frozen at −40°C.

Due to the influence of the isotopic composition of water on the biological properties and total extra-activity of proteins and peptides, water with deuterium concentration of 50 ppm was used for the extraction [12]. Molecular composition was characterized as described by Fedulova *et al*. [13], including α-thymosin (C3VVV8_PIG), rho GDP-dissociation inhibitor 1 (ARHGDIA)+ Acetyl (Protein N-term), rho GDP-dissociation inhibitor 2 (ARHGDIB), serpin B9 (SERPINB9), tyrosine kinase-binding protein (TYRO) and calnexin (CNX), C-terminus cathepsin S (STSS), myeloid differentiation primary response protein (MyD88), and transgelin (TAGLN), among others.

Tissue-specific biomolecules immobilized into alginate capsules were prepared on the basis of the technology developed by Manjanna *et al*. [17]. In brief, sodium alginate (Ruskhim, Russia) was dissolved in distilled water containing protein-peptide complexes (with a protein concentration in the solution of 21±2 g/L) until a final concentration of alginate of 1.2% by weight was achieved using a laboratory dispersion unit (Laboteks, Russia). The resulting suspension was passed using a peristaltic pump (Shenchenlab 2015 MCSeries, China) with a tube diameter of 1.5 mm and nozzle with a hole diameter of 0.15 mm in a 2% solution of calcium chloride (AppliChem Panreac, Germany) with a final pH of the solution equal to 4.0 (adjustment to the target pH was performed with 1 M NaOH). Next, the obtained capsules that were continuously mixed for 30 min on an MM-5 magnetic stirrer (Russia), washed with distilled water, and freeze-dried (INEY-4, Russia) at a temperature of −40±2°C and pressure of 3.3 kPa.

### Experimental plan

The study on pigs was performed in the Krolinfo LLC laboratory animal resource Center (NoLikino-Dulyovo, Russia). Twenty-five male Vietnamese pot-bellied×Wiesenau weaned pigs with an initial BW of 2.3±1.40 kg and initial age (weaning age) of 25-30 days were used for a 4-week experiment. Piglets were labeled with ear tags with numbers from 1 to 25. Weighing was performed with electronic floor scales MIDL “Gulliver 12” (MIDLiK, Russia) [18].

For this study, two groups of piglets were formed: Low-weight piglets (less than 2.0 kg) were selected into the experimental group and larger piglets (more than 2.0 kg) were selected into the control group. Pigs of the experimental group (n=15, 1.70 [1.37-1.85] kg) were individually treated orally for 28 days with encapsulated biomolecules at a dose of 150.0 mg capsules per kg BW. Pigs in the control group (n=10, 2.72 [2.22-2.95] kg) were treated with an equivalent volume of water for 28 days.

The piglets were housed individually in an environmentally controlled nursery with hard plastic slatted flooring and fed *ad libitum*. The pig houses were kept clean and all the piglets were of a healthy status. Experimental animals were all negative for relevant infections (*Bordetella bronchiseptica*; *Leptospira* spp.; *Pasteurella multocida*; *Salmonella* spp.; *Streptococci beta-hemolutic*; *Escherichia coli*; and *Toxoplasma gondii*).

The temperature in the room throughout the experiment was 24±1°C. Throughout the experiment, all piglets were fed SK-7 full-feed compound (Russia), which contained barley (40.5%), wheat (26.0%), wheat bran (18.0%), sunflower meal (≤36% crude protein) (5.0%), wheat-based feed grain product (5.0%), soy protein concentrate (2.0%), limestone flour (2.0%), premix P52-1 (1.0%), and table salt (0.5%) (Table-1). The feed pellets were steamed in a mixture of milk and boiling water before each feeding.

**Table-1:** Nutrient specifications of piglet feed.

Item, unit	Amount	Vitamin, unit	Amount	Mineral, mg/kg	Amount
Crude protein, %	15.00	A, UI/kg	3000	Iron	5.00
Crude fiber, %	7.00	D3, UI/kg	500	Manganese	3.00
Lipid, %	2.90	E, mg	3.0	Zinc	22.00
Available lysine, %	0.45	B2, mg	3.0	Cooper	6.00
Available methionine + cystine, %	0.38	B3, mg	7.0	Cobalt	0.50
		B5, mg	385	Iodine	0.40
Digestible energy, kcal/g	2.52	B12, mg	0.025	Selenium	0.15

Weighing and veterinary examination of animals was performed on days 0, 7, 14, and 21. Blood samples for studies were taken in the morning from the jugular vein on days 0, 14, and 28 before feeding.

### Hematology assays

A general clinical analysis of whole blood was performed on an Abacus Junior Vet 2.7 automatic veterinary hematology analyzer (Diatron Messtechnik GmbH, Austria). The following blood parameters were assayed: white blood cell count, red blood cell (RBC) count, hemoglobin (HGB), hematocrit (HCT), platelet (PLT), plateletcrit (PCT), and mean platelet volume (MPV) [15].

### Flow cytometry

The functional activity of leukocytes (relative content of lymphocytes, granulocytes, and monocytes) was determined on a Guava easyCyte automatic flow cytometer (Merck Millipore, Germany) [15].

### Biochemical assays

The pig blood serum biochemical parameters, including total protein (TP), albumin (A), creatinine (Cr), urea (U), direct bilirubin (DBr), aspartate aminotransferase (AST), alanine transaminase (ALT), lactate dehydrogenase, gamma-glutamyltransferase (GGT), alkaline phosphatase (ALP), triglyceride (TGr), total cholesterol (TC), and glucose (G), were determined on a BioChem FC-360 automatic biochemical analyzer (HTT, USA) using reagent kits (HTT, USA) [19].

### Enzyme-linked immunosorbent assay (ELISA)-tests

An ELISA was performed on an Immunochem 2100 analyzer (USA) using the sandwich method with ELISA species-specific reagent kits (pigs) in order to cytokines interleukin (IL)-2 and IL-4 [15].

### Blood serum antimicrobial activity (BSAA)

To study the animal resistance on 28^th^ day of the experiment, the integral index of BSAA was determined using a culture of *E. coli*. *E. coli* (24 h culture) preparation, which consists of a dilution to 2×10^9^ CFU in 1 mL of sterile saline solution (ZOMZ, Russia), followed by addition of 10 μL of the microbial suspension obtained to 4.5 μL of meat–peptone broth and incubation for 24 h (daily culture broth) [20].

Into sterile tubes containing 4.5 μL of meat–peptone broth, 1 mL of test serum was added. Next, 0.1 mL of *E. coli* (24 h culture) was added to all tubes; 2 mL was taken from each sample; and the optical density was measured at 420 nm. The mixture remaining in the tubes was incubated at 37°C for 180 min. Next, 2 mL was also taken from each sample and the absorbance was measured at 420 nm. The control was a test tube with serum-free meat and peptone broth. BSAA was expressed in growth inhibition units (conventional units) in accordance with the formula:

BSAA = 100×(E_op3_−E_op0_)/(E_k3_–E_k0_),

Where E_op0_ is the optical density of the experimental sample before incubation;

E_op3_ is the optical density of the experimental sample after 3 h of incubation;

E_k0_ is the optical density of the control sample before incubation;

E_k3_ is the optical density of the control sample after 3 h of incubation.

### Statistical analysis

Statistical processing of the results was performed with STATISTICA software program Versions 10.0 (Dell, USA). Intergroup comparison was performed by non-parametric Mann–Whitney test, with pairwise comparison within groups done by Wilcoxon signed-rank test. Results are presented as median (Me) and interquartile range ([P25-P75]) [21].

## Results

Despite the significantly lower mass of Group 2 animals relative to control animals at the zero-point (1.6 times at p<0.001), on the 28^th^ day, the piglets did not differ significantly in weight (Table-2). Experimental animals of both groups gained weight stably for 4 weeks. The weight of piglets in the 1^st^ and 2^nd^ weeks of the experiment was 2.72 (2.22-2.95) kg and 2.90 (2.63-3.20) kg for Group 1 and 1.70 (1.37-1.85) kg and 1.98 (1.61-2.13) kg for Group 2. In weeks 3 and 4, the animals’ weight was up to 3.81 (2.97-5.14) kg and 3.96 (3.64-5.57) kg in Group 1; however, in Group 2, there was a sharp increase in weight to 2.96 (2.37-3.49) kg and 3.37 (3.05-4.44) kg (33.1% at p<0.001 and 12.2% at p=0.064). High weight gain in week 3 caused the increased final weight gain in Group 2 animals, as a result of which their mass reached the values of piglets in the control group.

**Table-2:** BW and weight gain of piglets (Me [P25-P75]).

Group	Initial BW, kg	Final BW, skg	Weekly weight gain, %			Final weight gain, %

			1	2	3	
1	2.54 (2.06-2.64)	3.96 (3.64-5.57)	7.13 (6.50-13.64)	9.07 (6.40-13.64)	31.57 (15.53-43.90)	69.79 (53.70-91.17)
2	1.54* (1.24-1.66)	3.37^ns^ (3.05-4.45)	15.29* (12.33-19.04)	17.32* (12.69-20.72)	73.13^ns^ (11.49-127.44)	150.85 (72.35-285.63)

* p<0.05 compared to group 1. BW=Body weight, ns=Non-significant

In Group 1 animals, following the analysis of the hematological parameters on the 14^th^ day relative to day 0, a 40% decrease in PLT, 35.6% decrease in PCT, and 16.3% decrease in MPV, were observed. On the 28^th^ day, relative to the 14^th^ day, a 21.2% increase in HGB was observed.

Moreover, in Group 2 animals, on the 14^th^ day relative to day 0, a 25.1% increase in erythrocyte level, 29.1% increase in HGB, and 23.9% increase in HCT, were observed (Tables-3 and 4); PLT decreased by 25.1% and PCT decreased by 31.9%. On the 28^th^ day, PLT continued to decrease by 34.4% relative to day 14 and PCT also decreased by 34.7%.

**Table-3:** Hematological parameters and serum biochemistry of experimental piglets (Me).

Parameter	Group	Control points			p-value	
	
		T0 (0 days)	T1 (14 days)	T2 (28 days)	T0 versus T1	T1 versus T2
White blood cells, 10^9^/L	1	23.17	25.16	26.38	0.100	0.433
	2	20.14	25.65	30.22	0.069	0.204
Red blood cells, 10^12^/L	1	7.12	6.84	6.27	0.167	0.161
	2	5.19	6.93	6.68	<0.001	0.424
Hemoglobin, g/L	1	87.50	104.00	111.00	0.126	<0.001
	2	79.00	111.50	114.50	<0.001	0.416
Hematocrit, %	1	22.66	27.67	28.88	0.279	0.073
	2	22.33	29.33	30.64	0.004	0.228
Platelets, 10^9^/L	1	837.01	502.50	485.50	0.007	0.218
	2	819.50	614.00	403.00	<0.001	0.003
Plateletcrit, %	1	0.59	0.38	0.37	0.007	0.296
	2	0.72^ns^	0.49*	0.32^ns^	0.004	0.014
Mean platelet volume, μm^3^	1	8.01	6.70	7.20	0.014	0.647
	2	6.80^ns^	7.95*	7.45^ns^	0.315	0.949
Total protein, g/L	1	62.10	59.00	55.68	0.028	0.465
	2	64.90^ns^	61.00^ns^	62.90*	0.005	0.889
Albumin, g/L	1	43.20	35.75	30.95	0.013	0.109
	2	46.40*	40.75*	37.18*	0.002	0.176
Creatinine, μM	1	64.05	49.25	51.38	0.005	0.465
	2	57.80	52.30	54.75	0.023	0.093
Urea, μM	1	7.75	3.96	3.05	0.005	0.465
	2	7.81^ns^	4.78*	4.96*	0.001	0.069
Direct bilirubin, μM	1	2.56	2.08	2.26	0.114	1.000
	2	4.41*	3.20*	2.45^ns^	0.156	0.401
Aspartate aminotransferase, Е/L	1	28.55	35.45	36.40	0.203	1.000
	2	45.10*	28.20^ns^	46.65^ns^	0.027	0.208
Alanine transaminase, Е/L	1	26.25	34.70	28.05	0.114	0.273
	2	47.30*	27.40^ns^	26.43^ns^	<0.001	0.093
Lactate dehydrogenase, Е/L	1	463.40	459.80	522.40	0.646	0.273
	2	587.20*	439.1^ns^	539.60^ns^	0.005	0.017
Gamma-glutamyltransferase, Е/L	1	41.34	30.48	26.11	0.013	0.068
	2	41.57^ns^	42.96^ns^	37.95*	0.691	0.484
Alkaline phosphatase , Е/L	1	317.35	150.55	124.35	0.007	0.715
	2	432.05^ns^	165.10^ns^	250.95*	0.001	0.025
Triglyceride, M	1	0.27	0.46	0.72	0.333	0.144
	2	0.45*	0.54^ns^	1.13*	0.426	0.012
Total cholesterol, M	1	8.41	5.56	2.24	0.007	0.144
	2	7.32^ns^	5.30^ns^	3.08*	<0.001	0.093
Glucose, mM	1	7.45	1.98	3.40	0.005	0.273
	2	7.50	2.39	4.75	0.036	0.933

* p<0.05 compared to Group 1. ns=Non-significant

**Table-4:** Hematological parameters and serum biochemistry of experimental piglets (P25-P75).

Parameter	Group	Control points			p-value	
	
		T0 (0 days)	T1 (14 days)	T2 (28 days)	T0 versus T1	T1 versus T2
White blood cells, 10^9^/L	1	20.44-26.17	19.83-30.24	22.91-33.66	0.100	0.433
	2	17.03-24.26	21.07-28.45	25.17-31.65	0.069	0.204
Red blood cells, 10^12^/L	1	4.27-7.71	6.32-7.33	5.72-7.14	0.167	0.161
	2	4.49-6.43	6.31-7.40	5.94-7.60	<0.001	0.424
Hemoglobin, g/L	1	79.75-108.25	93.50-113.00	98.75-119.25	0.126	<0.001
	2	63.00-95.50	102.00-121.50	102.50-126.00	<0.001	0.416
Hematocrit, %	1	20.81-28.47	24.03-31.87	25.70-31.28	0.279	0.073
	2	17.83-26.78	25.94-33.49	26.86-35.46	0.004	0.228
Platelets, 10^9^/L	1	566.75-1315.50	403.50-725.50	291.75-771.50	0.007	0.218
	2	607.00-2266.00	533.25-928.50	295.00-618.75	<0.001	0.003
Plateletcrit, %	1	0.37-1.26	0.30-0.46	0.19-0.55	0.007	0.296
	2	0.35-2.36^ns^	0.39-0.68*	0.22-0.50^ns^	0.004	0.014
Mean platelet volume, μm^3^	1	6.28-10.63	6.20-7.60	6.35-7.73	0.014	0.647
	2	6.30-11.35^ns^	6.83-8.38*	7.13-8.10^ns^	0.315	0.949
Total protein, g/L	1	60.43-69.03	57.38-60.83	51.48-58.68	0.028	0.465
	2	63.65-68.65^ns^	58.98-64.00^ns^	59.40-65.48*	0.005	0.889
Albumin, g/L	1	41.70-45.88	34.18-38.88	29.88-32.10	0.013	0.109
	2	44.80-49.45*	39.15-42.48*	36.65-38.55*	0.002	0.176
Creatinine, μM	1	58.30-68.45	43.08-53.58	47.92-55.31	0.005	0.465
	2	56.35-64.35	48.70-55.05	53.43-60.70	0.023	0.093
Urea, μM	1	6.18-15.16	3.30-4.50	2.89-3.24	0.005	0.465
	2	6.12-13.03^ns^	3.86-5.16*	4.66-5.33*	0.001	0.069
Direct bilirubin, μM	1	2.07-3.10	1.78-2.40	1.95-2.84	0.114	1.000
	2	3.73-5.89*	2.47-4.83*	2.01-2.73^ns^	0.156	0.401
Aspartate aminotransferase, Е/L	1	25.40-43.60	31.55-47.48	27.30-48.88	0.203	1.000
	2	39.60-58.35*	26.30-36.40^ns^	35.78-57.53^ns^	0.027	0.208
Alanine transaminase, Е/L	1	22.90-33.48	32.55-40.30	27.53-34.30	0.114	0.273
	2	37.75-49.50*	23.20-44.60^ns^	25.58-31.23^ns^	<0.001	0.093
Lactate dehydrogenase, Е/L	1	458.83-530.33	411.03-575.88	411.93-624.48	0.646	0.273
	2	532.05-719.70*	394.25-521.45^ns^	525.35-638.90^ns^	0.005	0.017
Gamma-glutamyltransferase, Е/L	1	39.50-44.37	29.03-38.18	24.33-29.00	0.013	0.068
	2	36.86-45.46^ns^	34.50-52.67^ns^	32.56-41.09*	0.691	0.484
Alkaline phosphatase, Е/L	1	204.98-441.95	111.65-166.80	108.13-141.75	0.007	0.715
	2	279.30-507.18^ns^	139.90-211.10^ns^	188.93-308.38*	0.001	0.025
Triglyceride, M	1	0.24-0.46	0.36-0.58	0.62-0.85	0.333	0.144
	2	0.38-0.69*	0.44-0.66^ns^	1.06-1.23*	0.426	0.012
Total protein, M	1	5.72-11.33	4.98-6.18	2.09-2.43	0.007	0.144
	2	5.95-9.86^ns^	4.50-5.65^ns^	2.67-3.46*	<0.001	0.093
Glucose, mM	1	7.05-8.35	1.85-2.21	3.05-3.98	0.005	0.273
	2	5.40-7.90	2.06-2.78	4.55-5.33	0.036	0.933

* p<0.05 compared to Group 1. ns=Non-significant

There were marked changes in animal blood serum biochemical markers, which characterize protein metabolism, on the 14^th^ day of the experiment. In Group 1, a 5.0% decrease in TP, 17.2% in A, 23.1% in Cr, and 48.9% in U, was observed. In Group 2 animals, a similar trend was noted, as a 6.0% decrease in TP, 12.2% in A, 9.5% in Cr, and 38.8% decrease in U was observed. When comparing these indicators on the 14^th^ and 28^th^ days, no significant changes were noted; however, a tendency of increased Cr and U in Group 2 animals was revealed.

Intergroup comparison in Group 2 animals on the 14^th^ and 28^th^ days revealed significant increases in A by 12.3% and 16.8% as well as in U by 17.1% and 38.5%, respectively. TP also increased on the 28^th^ day by 11.5%.

For enzyme parameters in Group 1, on the 14^th^ day, the activity of GGT decreased by 26.3% and that of ALP decreased by 52.6%. In Group 2 animals, on the 14^th^ day, activity of AST decreased by 37.5%, ALT by 42.1%, LDH by 25.2%, and ALP by 61.8%. On the 28^th^ day, relative to the 14^th^ day, the activity of LDH and ALP increased by 22.9% and 52.0%, respectively.

An intergroup comparison revealed that, in Group 2 animals, at the commencement of the experiment (0 day), DBr was increased by 41.9%, AST by 36.7%, ALT by 44.5%, and LDH by 21.1%. On day 14, in Group 2, DBr was increased by 35.0%; on the 28^th^ day, GGT and ALP activity increased by 31.2% and 50.4%, respectively.

Changes were also noted among the parameters of lipid metabolism. On day 14, Groups 1 and 2 showed a decrease in TC by 33.9% and 27.6%, respectively; on the 28^th^ day, TGr in Group 2 animals increased by 52.2%. An intergroup comparison in Group 2 was done when the experiment had just begun and a 40% increase in TGr was found. On the 28^th^ day, an increase in TGr (36.3%) and TC (27.3%) was noted.

**Figure-1:**
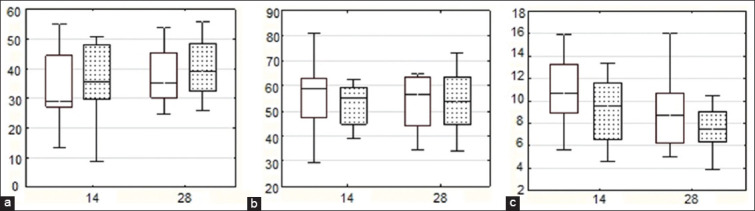
Relative content of lymphocytes (LIM), granulocytes (GRAN), and monocytes (MON) in piglet blood on the 14^th^ and 28^th^ days: (a) LIM, %; (b) GRAN, %; (c) MON, %. Data presented as box plots include hinges extending from the 25^th^ to 75^th^ percentiles, the median line within the box and whiskers extending to the minimum and maximum values of the dataset. Group 1 – white boxes, Group 2 – boxes with black dots.

Furthermore, G in the blood serum of animals of both groups on the 14^th^ day, relative to day 0 was decreased by 73.4% and 68.1%, respectively. Animal blood leukocyte typing revealed the absence of significant changes both in the intergroup comparison and in comparison of parameters on the 14^th^ and 28^th^ days (Figure-1).

ELISA test of cytokine level in Group 2 animals’ blood serum revealed a 70.2% increase in IL-2 level on the 14^th^ day relative to day 0 and a 25.2% increase on the 28^th^ day relative to the 14^th^ day. IL-4 level increased on the 28th day relative to the 14^th^ day by 82.0% (Table-5).

**Table-5:** Immunoassay analysis of cytokines (Me [P25-P75]).

Parameter	Group	Control points			p-value	
	
		T0 (0 days)	T1 (14 days)	T2 (28 days)	T0 versus T1	T1 versus T2
IL-2, pg/mL	1	634.27 (292.98-815.98)	795.72 (709.79-1217.10)	946.29 (647.82-2093.14)	0.114	0.333
	2	352.02 (199.02-493.32)	1182.24 (847.92-1611.18)	1580.07 (1158.58-2505.14)	0.001	0.023
IL-4, pg/mL	1	63.14 (29.44-78.45)	42.14 (33.03-49.09)	41.37 (32.60-104.54)	0.203	0.333
	2	18.90 (13.14-35.84)	21.01 (19.55-33.64)	116.83 (54.53-180.02)	0.311	0.002

IL=Interleukin

At the end of the experiment, BSAA index was 71.03 (62.15-82.24) units for Group 1 animals and 52.34 (50.47-53.27) units for Group 2 animals, thus indicating increased bactericidal activity in Group 2 animals’ blood serum.

## Discussion

The use of antibiotics often leads to the development of side effects and spread of antibiotic-resistant diseases. This study investigated the effectiveness of natural immunomodulatory biomolecules included in alginate capsules.

The study results prove the efficiency of encapsulated biomolecules as an immunomodulator. Experimental piglet-weaners reached the control animals’ weight in 28 days, thus overcoming the initial weight difference (by 1.6 times) [22]; moreover, there were increases in blood component antimicrobial activity (including BSAA) and cytokine production as well as the absence of pronounced changes in the immune system [23]. The clinical experiment showed an increase in the content of IL-2 and IL-4 as well as antimicrobial activity with stable levels of leukocytes and young immune cells, thus indicating an increase in functional activity, first of all, in the humoral immune system [24].

In addition, in this study, the increases in RBC and HGB at the 28^th^ day of the experiment in Group 2 are similar to the values reported in other publications [25,26] and it correlates with a weight gain increase of more than 70% at 3 experimental weeks. Changes in PLT and erythrocyte markers for piglets, accompanied by individual parameter variability in each experimental group, especially on days 0-14, which is reflected in a wide interquartile range, are associated with intensive growth of animals and maturation of their hematopoietic system. The decrease in PLT count in piglets in our study, starting from the 2^nd^ experimental week, is consistent with the observations of other studies [27,28]. This trend may indicate an increase in the production of large PLTs by bone marrow megakaryocytes due to the activation of thrombopoiesis by biomolecules [29].

In this study, we found that the use of biomolecules for weaned piglets had a positive effect on protein synthesis in the liver, as evidenced by TP and A levels in serum normalization. According to Yu *et al.*, this is the result of adaptation of animal protein metabolism in connection with the transition from breast milk to animal feed and because it is also associated with the rapid growth of piglets, leading to a rapid increase in muscle mass. In their article, they demonstrated similar changes in protein metabolism in recently weaned piglets [30]. A decrease in enzyme activity indicated by hepatic markers, coupled with a decrease in protein and Cr levels, can reflect the metabolic status of hepatic protein in response to dietary changes in weaned piglets [31].

According to a previous study [32], an increase in ALP concentration in young animals is probably associated with higher osteoblast activity; however, this effect needs to be clarified.

As established in the control animals, hypoproteinemia, hypoalbuminemia, hypoglycemia, hypoureaemia, hypercreatininemia, and hyperbilirubinemia with a decrease in blood enzyme activity indicate the difficulty of adaptation of piglets’ metabolic processes during ration changing to solid food (animal feed). Biomolecules had a positive effect on the gluconeogenesis process in pigs, as evidenced by an increase in serum G levels; moreover, there was a net positive influence on the pigment-forming function of the liver and functioning of the liver and biliary tract, as evidenced by a decrease in total bilirubin and activity of the aminotransferases ALT and AST as well as and ALP and GGT.

## Conclusion

The antimicrobial activity of the blood serum increased significantly after taking the immunomodulator. The level of cytokines involved in the immunity of piglets also increased. In general, the immunomodulator favorably affects the metabolism of animals and reduces exposure to stress. It was shown that biomolecules can cause a positive effect on the adaptation of piglets’ metabolism during transition from colostrum to solid food by improving the functional activity of the liver, particularly by increasing protein synthesis.

## Authors’ Contributions

ERV and LVF were responsible for conception and design of the study. EAK and AIF coordinated the analysis. IMC was responsible for the conclusive and final remarks. ERV and LVF did the final editing and approval along with researchers. All authors read and approved the final manuscript.
